# Isolation, molecular characterization and prevalence of *Clostridium perfringens* in sheep and goats of Kashmir Himalayas, India

**DOI:** 10.14202/vetworld.2017.1501-1507

**Published:** 2017-12-22

**Authors:** Salik Nazki, Shakil A. Wani, Rafia Parveen, Showkat A. Ahangar, Zahid A. Kashoo, Syed Hamid, Zahoor A. Dar, Tanveer A. Dar, Pervaiz A. Dar

**Affiliations:** 1 Bacteriology Laboratory, Division of Veterinary Microbiology and Immunology, Faculty of Veterinary Sciences and Animal Husbandry, SKUAST-Kashmir, Shuhama, Srinagar - 190 006, Jammu and Kashmir, India; 2 Laboratory of Veterinary Immunology, College of Veterinary Medicine, Chonbuk National University, Iksan 54596, South Korea; 3 Bacteriology Laboratory, Division of Veterinary Microbiology and Immunology, Faculty of Veterinary Sciences and Animal Husbandry, SKUAST-Jammu, R.S. Pora, Jammu - 181 102, India; 4 Division of Veterinary Pathology, Faculty of Veterinary Sciences and Animal Husbandry, SKUAST-Kashmir, Shuhama, Srinagar - 190 006, Jammu and Kashmir, India

**Keywords:** *Clostridium perfringens*, enterotoxemia, multiplex polymerase chain reaction, toxinotype, 16S rRNA

## Abstract

**Aim:**

The study was conducted to report the occurrence of the *Clostridium perfringens* in sheep and goats of the Kashmir valley for the 1^st^ time and to characterize them molecularly with respect to toxin genes to determine the prevalence of the various toxinotypes.

**Materials and Methods:**

A total of 177 samples (152 from sheep and 25 from goats) collected from healthy, diarrheic animals, and morbid material of animals suspected to have died of enterotoxaemia were screened for *C. perfringens* toxinotypes. The presumptive positive isolates were confirmed using 16S rRNA gene-based polymerase chain reaction (PCR). All the confirmed isolates were screened for six toxin genes, namely; *cpa, cpb, etx, cpi, cpb2*, and *cpe* using a multiplex PCR.

**Results:**

The PCR amplification of 16S rRNA gene revealed that out of 177 samples collected, 125 (70.62%) were found positive for *C. perfringens*, of which 110 (72.36%) were from sheep and 15 (60%) were from goats. The highest prevalence of *C. perfringens* toxinotype D was observed in lambs (56.16%) and kids (46.16%) followed by 3.84% in adult sheep while it was absent in samples obtained from adult goats. The multiplex PCR revealed that 67 (60.90%) isolates from sheep and 8 (53.33%) isolates from goats belonged to toxinotype A, while 43 (39.09%) isolates from sheep and 7 (46.66%) isolates from goats were detected as toxinotype D. None of the isolates was found to be toxinotype B, C, or E. All the *C. perfringens* toxinotype A isolates from sheep were negative for both *cpb2* and *cpe* genes, however, 27.90% toxinotype D isolates from sheep carried *cpb2* gene, and 6.97% possessed *cpe* gene. In contrast, 12.50% *C. perfringens* toxinotype A isolates from goats harbored *cpb2* and *cpe* genes while 14.28% isolates belonging to toxinotype D carried *cpb2* and *cpe* genes, respectively.

**Conclusion:**

The high prevalence of *C. perfringens* was observed, even in day-old lambs. The toxinotypes A and D are prevalent in both sheep and goats. The severity of disease and mortality may be associated with the presence of minor toxins in both the detected toxinotypes.

## Introduction

*Clostridium perfringens* is one of the ubiquitous organisms among clostridial species. It is the common inhabitant of the gastrointestinal tract of humans and animals and also occurs in the soil. It is relatively aero-tolerant, spore-forming, non-motile, and Gram-positive rods (0.6-0.8×2-4 µm) [1]. On the basis of four major toxins, namely, alpha (CPA), beta (CPB), epsilon (ETX), and iota (ITX), the *C. perfringens* is divided into five toxinotypes, i.e. A, B, C, D, and E [2,3]. The specific toxins are responsible for the clinical signs and a syndrome attributable to each type. The specific enteric infections of various animal species are associated to different toxinotypes [4,5].

All the five toxinotypes produce alpha toxin and in addition to that toxinotype B produces beta and epsilon-toxin while toxinotype C produces beta-toxin, toxinotype D produces epsilon-toxin, and toxinotype E strains produce iota-toxin. Along with major toxins, *C. perfringens* toxinotypes also produce some minor toxins such as enterotoxin (CPE) and beta2 toxin (CPB2) [6]. Isolates of *C. perfringens* carrying the gene for beta2 toxin have been identified, suggesting a role for beta2-toxin in caprine enterotoxemia and a common cause of death in growing kids [7,8]. The *C. perfringens* toxinotype D is of utmost ­importance in case of sheep and goats, responsible for ovine enterotoxemia and caprine enterocolitis, respectively. Enterotoxemia is an acute, highly fatal intoxication that affects sheep, lambs, kids, and goats [9]. Sheep of all ages are affected by enterotoxemia, but lambs under 10 weeks of age are most susceptible as they are nursed by heavy-lactating ewes and the weaned lambs on lush pasture or in feedlots [10,11].

Enterotoxemia results in the colossal economic losses to the farming industry worldwide [12]. In 2010-2011, livestock generated a total of 4% of the gross domestic product (GDP) and 26% of the agricultural GDP in India. Sheep rearing is considered to be one of the major contributors to the livestock sector. The economics of sheep farming depends largely on the survival of the lambs and later lambing percentage of adult stock. A study showed that enterotoxemia (incidence rate - 1.5%, death rate - 2.4%, and case fatality rate - 30.8%) comes next to diseases such as bluetongue, PPR, and anthrax with respect to incidence and death rate in India [13].

The present study was conducted for a period of 6 months (October 2014 to April 2015) to investigate the prevalence of *C. perfringens*, in sheep and goats of Kashmir valley as well as characterize the genotype of its isolates. In spite of its heavy impact on sheep rearing farms, there seems no literature available on any aspect of *C. perfringens* toxinotype D in the Kashmir Himalayas, India. This study documented the presence of *C. perfringens* toxinotype A and D in sheep and goats for the 1^st^ time in Kashmir.

## Materials and Methods

### Ethical approval

The approval from the Institutional Animal Ethics Committee to carry out this study was not required as no invasive technique was used. The fecal samples were collected from healthy, clinically affected with enterotoxemia and dead animals as per standard collection procedure.

### Samples

A total of 177 samples comprising fecal material, and intestinal contents, kidneys, and abomasum pieces from animals suspected to have been died due to enterotoxemia were collected from 152 sheep and 25 goats. The samples were collected from organized sheep farms and unorganized sectors such as local farmers rearing sheep and goats. Out of the 152 samples of sheep, fecal material was collected from healthy (125) and diarrheic (21) sheep while kidneys, abomasum pieces and intestinal contents were collected from 6 carcasses. Among goats, fecal samples were collected from healthy (20) and diarrheic (3) goats while kidneys and intestinal contents were collected from 2 dead animals. The samples were collected within 2 hours after death of animals. The samples were collected in sterile vials from animals of different age groups.

### Isolation and identification of *C. perfringens*

For isolation of *C. perfringens*, samples were inoculated in Difco^™^ Cooked meat medium (Becton, Dickinson and Company, Sparks, MD, USA) and incubated anaerobically in 3.5 litre anaerobic jar (Oxoid Limited, Thermo Fisher Scientific Inc., UK) with GasPak^™^ Anaerobe Container System (Becton, Dickinson and Company, Sparks, MD, USA) at 37°C for 24 h. Enriched samples were streaked on Sulfite Polymyxin Sulfadiazine agar plates (SPS HiVeg^™^ Agar, Modified; HiMedia laboratories, Mumbai, India) and the plates were incubated anaerobically at 37°C for 24 h. After incubation suspected colonies were sub-cultured on the SPS agar plates until they were free from contaminating bacteria. The pure cultures of *C. perfringens* toxinotypes were lyophilized for future use in the laboratory using 0.25 M sucrose as a cryoprotectant.

Confirmation of the isolates was done by demonstration of the typical cellular morphology in Gram’s stained smear, standard biochemical tests [14] and detection of *C. perfringens* by species-specific polymerase chain reaction (PCR) using 16S rRNA gene primers [15,16].

### Molecular characterization of *C. perfringens* isolates

#### Bacterial DNA isolation

Suspected isolated colonies from agar plates were suspended in 1.5 ml microcentrifuge tubes containing 100 μl of distilled water by gentle vortexing. The samples were boiled for 5 min, cooled on ice for 10 min and centrifuged at 10,000×*g* in a table-top microcentrifuge (Cooling Centrifuge, Eppendorf 5418R, Hamburg, Germany) for 1 min. 3 microliters (μl) of the supernatant were used as the template for PCR.

#### PCR

All the PCR assays in this study were performed in 25 µl reaction volume in Mastercycler gradient (Eppendorf AG, Hamburg, Germany). The reaction consisted of 3.0 µl template DNA, 2.5 µl of 10× buffer, 0.2 µl of 25 mM dNTP mix, 1 U of Taq DNA Polymerase (Fermentas Life Sciences) and sterile distilled water. The MgCl_2_ was used at 2.0 mM concentration unless otherwise indicated. Sterilized distilled water was used as negative controls. All the primers were acquired from GCC Biotech, Kolkata, India.

#### 16S rRNA gene amplification

The species-specific primers (Table-1) [15,17] targeting 16S rRNA gene of the *C. perfringens* was used. The PCR conditions consisted of initial denaturation at 95°C for 15 min, followed by 35 cycles of denaturation at 94°C for 30 s, annealing at 49°C for 90 s, and extension at 72°C for 90 s. This was followed by final extension at 72°C for 10 min. The DNA of *C. perfringens* type D isolates obtained from Sheep Husbandry Department was used as positive control.

**Table-1:** List of primers used in PCR for amplification of *Clostridium perfringens* toxin genes.

Target gene	Primer sequence (5′-3′)	Primer conc. (µM)	Product size (bp)	References
16S rRNA	F-TAACCTGCCTCATAGAGT	0.4	481	[15]
	R-TTTCACATCCCACTTAATC			
*cpa*	F-GCTAATGTTACTGCCGTTGA	0.4	324	[17]
	R-CCTCTGATACATCGTGTAAG			
*cpb*	F-GCGAATATGCTGAATCATCA	0.4	195	[17]
	R-GCAGGAACATTAGTATATCTTC			
*etx*	F-TGGGAACTTCGATACAAGCA	0.4	376	[17]
	R-AACTGCACTATAATTTCCTTTTCC			
*cpi*	F-AATGGTCCTTTAAATAATCC	0.4	272	[17]
	R-TTAGCAAATGCACTCATATT			
*cpb2*	F-AAATATGATCCTAACCAACAA	0.4	548	[17]
	R-CCAAATACTCTAATYGATGC			
*cpe*	F-TTCAGTTGGATTTACTTCTG	0.4	485	[17]
	R-TGTCCAGTAGCTGTAATTGT			

PCR=Polymerase chain reaction

#### Multiplex PCR of virulent genes

The *C. perfringens* isolates were characterized for important virulence factors including *cpa, cpb*, *etx, cpi, cpb2*, and *cpe*. All the *C. perfringens* isolates were screened for these toxin genes using a multiplex PCR. These six toxin genes include α-toxin (*cpa*), β-toxin (*cpb*), ε-toxin (*etx*), Í-toxin (*cpi*), β2-toxin (*cpb-2*), and enterotoxin (*cpe*). The primers used for the amplification of the genes are shown in Table-1. The PCR conditions were similar to that used for amplification of 16S rRNA gene except for the annealing temperature that was set at 53°C. The amplified products were electrophoresed in 1.5% agarose gel (Sigma-Aldrich, St. Louis, USA) and stained with ethidium bromide (0.5 µg/ml). Amplified bands were visualized and photographed under UV illumination (Ultra Cam Digital Imaging, Ultra. Lum. Inc., Claremont, CA).

## Results

### Detection and prevalence of *C. perfringens* toxinotypes from samples

**Figure-1:**
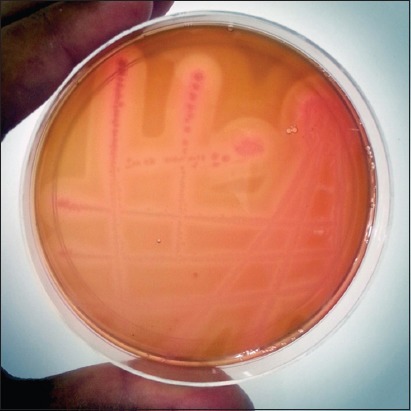
Double zone of hemolysis produced by *Clostridium perfringens* on sheep blood agar.

**Figure-2:**
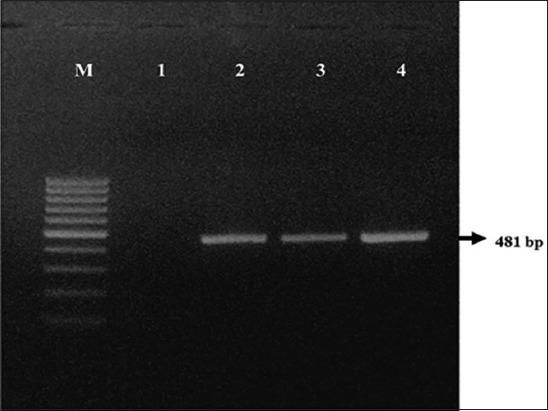
481 bp amplicon characteristic of 16S rRNA gene of *Clostridium perfringens*. Lane M: 100 bp DNA ladder. Lane 1: Negative control. Lane 2: Positive control. Lanes 3-4: Isolates positive for *C. perfringens*.

From 152 samples of sheep, 110 (72.36%) carried *C. perfringens* while, out of 25 samples from goats, 15 (60%) revealed the presence of *C. perfringens*. Out of 125 samples collected from healthy sheep, 86 (68.80%) were positive for *C. perfringens*, while 19 (90.47%) out of 21 enterotoxemia suspected live sheep and 5 (83.33%) out of 6 enterotoxemia suspected dead sheep were detected positive for *C. perfringens*. Among goats, 11 (55%) out of 20 healthy goats were detected positive for *C. perfringens* while 2 (66.66%) out of 3 diarrheic goats and both dead goats were positive for *C. perfringens*. All the 110 isolates from sheep and 15 isolates from goats were morphologically and biochemically identified by Gram staining, capsular staining, lecithinase activity on egg yolk agar media, triple sugar iron (TSI) test, and formation of double zone of hemolysis on 5% sheep blood agar (Figure-1) as *C. perfringens*. These isolates amplified 481bp PCR product (Figure-2) corresponding to *C. perfringens*.

**Figure-3:**
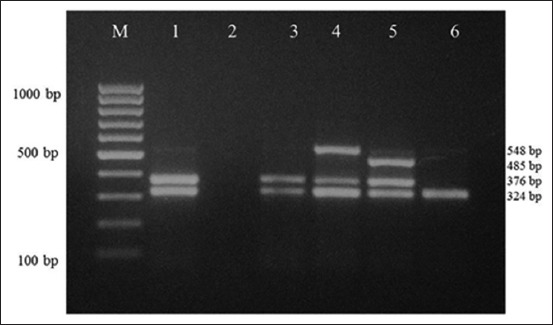
Multiplex polymerase chain reaction amplicons of different virulence genes of *Clostridium perfringens*. Lane M: 100 bp DNA ladder. Lane 1: Positive control of *C. perfringens* Type D with amplified *cpa* (324 bp) and *etx* (376 bp) genes. Lane 2: Negative control. Lane 3: *C. perfringens* Type D. Lane 4: *C. perfringens* Type D with amplified *cpa, etx* and *beta2 (*548 bp) genes. Lane 5: *C. perfringens* Type D with amplified *cpa, etx*, and *cpe* (485 bp) genes. Lane 6: *C. perfringens* Type A with *cpa* gene amplification.

Out of a total of 110 isolates from sheep, 67 (60.90%) were found to carry *cpa* gene alone as a major toxin gene, thus were designated as toxinotype A. While the remaining 43 (39.09%) harbored both *cpa* and *etx* genes, thus were designated as toxinotype D. Among 86 isolates from healthy sheep, 60 (69.77%) isolates were tested to be positive for toxinotype A while 26 (30.23%) were found to be toxinotype D. Out of total 24 isolates from sheep suffering diarrhea or suspected to have died of ­enterotoxemia, 7 (29.16%) isolates were found to be toxinotype A while 17 (70.84%) isolates were detected as toxinotype D (Table-2). *C. perfringens* toxinotype D was isolated from kidneys, intestinal contents and abomasum of 4 dead sheep while remaining 2 showed the presence of toxinotype A. None of the isolates carried *cpb* or *cpi* genes indicating the absence of *C. perfringens* toxinotype B, C, or E in sheep samples. In case of goats, out of 15 isolates, 8 (53.33%) were detected as toxinotype A as they carried *cpa* gene alone and the remaining 7 (46.66%) as toxinotype D which carried both *cpa* and *etx* genes. From 11 isolates of healthy goats, 6 (54.54%) isolates were detected as toxinotype A while 5 (45.46%) isolates were confirmed to be toxinotype D. Among 4 isolates from goats suffering diarrhea or suspected to have died of enterotoxemia, 2 (50%) were confirmed as toxinotype A and 2 (50%) as toxinotype D. *C. perfringens* toxinotype D was isolated from kidneys, intestinal contents and abomasum of both dead goats. None of the isolates possessed *cpb* or *cpi* genes indicating the absence of *C. perfringens* toxinotype B, C, or E in goat samples (Figure-3).

**Table-2:** *Clostridium perfringens* toxinotype distribution among healthy and enterotoxemia suspected sheep and goats.

Toxinotype	Species					

	Sheep		N	Goat		N
	
	ET suspected (%)	Healthy (%)		ET suspected (%)	Healthy (%)	
A	7 (29.16)	60 (69.77)	67 (60.91)	2 (50)	6 (54.54)	8 (53.33)
D	17 (70.84)	26 (30.23)	43 (39.09)	2 (50)	5 (45.46)	7 (46.67)
Total	24	86	110	4	11	15

Further, the sheep samples were collected from both organized as well as unorganized sectors. Out of the total of 89 isolates from organized sector, 48 (53.93%) isolates were detected as toxinotype A while the remaining 41 (46.06%) isolates were found as toxinotype D (Table-3). Among the 21 isolates from unorganized sector, 19 (90.47%) isolates were toxinotype A while 2 (9.52%) isolates were toxinotype D. The occurrence of toxinotype D was found to be higher in organized sector (46.06%) than that of unorganized sector (9.52%).

**Table-3:** Prevalence of *Clostridium perfringens* toxinotypes in sheep under organized and unorganized rearing conditions.

Sampling area	Total number of isolates	Toxinotypes	

		A N (%)	D N (%)
Organized sector	89	48 (53.93)	41 (46.06)
Unorganized sector	21	19 (90.47)	2 (9.52)
Total	110	67 (60.90)	43 (39.09)

### Identification of *C. perfringens* toxinotypes carrying minor toxin genes

In sheep, among *C. perfringens* toxinotype A isolates none of the isolates carried *cpb2*-gene or *cpe*-gene in addition to the species-specific *cpa* gene. Among *C. perfringens* toxinotype D isolates, 12 (27.90%) were positive for *cpb2* and 3 (6.97%) harbored *cpe*-gene in addition to the *cpa* and *etx* genes. Out of 24 isolates from sheep suspected of enterotoxemia, 4 (16.66%) carried *cpb2* while 2 (8.34%) were detected positive for *cpe* gene in toxinotype D. Among healthy sheep, 8 (9.30%) carried cpb2 while 1 (1.17%) isolate harbored cpe gene in toxinotype D out of 86 isolates (Table-4).

**Table-4:** Distribution of major and minor toxin genes in *Clostridium perfringens* toxinotypes isolated from healthy and enterotoxemia suspected sheep and goats.

Toxinotype	Species							

	Toxin genes	Sheep			Goat		
	
		ET suspected (%)	Healthy (%)	N (%)	ET suspected (%)	Healthy (%)	N (%)
A	*cpa*	7 (29.16)	60 (69.77)	67 (100)	0 (0.00)	6 (54.54)	6 (75.00)
	*cpa* and *cpb2*	0 (0.00)	0 (0.00)	0 (0.00)	1 (25.00)	0 (0.00)	1 (12.50)
	*cpa* and *cpe*	0 (0.00)	0 (0.00)	0 (0.00)	1 (25.00)	0 (0.00)	1 (12.50)
	N			67			8
D	*cpa* and *etx*	11 (45.84)	17 (19.76)	28 (65.11)	2 (50.00)	3 (27.28)	5 (71.42)
	*cpa*, *etx* and *cpb2*	4 (16.66)	8 (9.30)	12 (27.91)	0 (0.00)	1 (9.09)	1 (14.28)
	*cpa*, *etx* and *cpe*	2 (8.34)	1 (1.17)	3 (6.98)	0 (0.00)	1 (9.09)	1 (14.28)
	N	24	86	43	4	11	7

In case of goats among *C. perfringens* toxinotype A, one (12.50%) isolate each carried either *cpb2* gene or *cpe*-gene in addition to *cpa* gene. Both these isolates were acquired from samples of goats suspected of suffering enterotoxemia. Among *C. perfringens* toxinotype D isolates, one (14.28%) isolate was positive for *cpb2* and one (14.28%) harbored *cpe*-gene in addition to the *cpa* and *etx* genes. Isolates carrying *cpb2* and *cpe* gene were detected in healthy goats.

Among lambs, the occurrence of toxinotype D (56.16%) was higher than that (28.76%) of toxinotype A (Table-5). In case of weaners, 55.55% carried toxinotype A while none of the samples from weaners revealed the presence of toxinotype D of *C. perfringens*. Among adult sheep occurrence of toxinotype A (59.61%) was higher than that (3.84%) of toxinotype D.

**Table-5:** Age-wise distribution of *Clostridium perfringens* toxinotypes in sheep and goats.

Species	Age	Total samples	Toxinotypes	

			A N (%)	D N (%)
Sheep	Lambs (0-3 months)	73	21 (28.76)	41 (56.16)
	Weaners (3-12 months)	27	15 (55.55)	0 (0.00)
	Adults (1 year or more)	52	31 (59.61)	2 (3.84)
Goats	Kids (0-6 months)	13	2 (15.38)	6 (46.16)
	Adults (more than 6 months)	12	7 (8.33)	0 (0.00)

In case of kids occurrence of toxinotype D (46.16%) was higher than that (15.38%) of toxinotype A. In case of adult goats 58.33% carried toxinotype A while none of the samples from adult goats revealed presence of toxinotype D of *C. perfringens*.

## Discussion

*C. perfringens* toxinotypes are responsible for varied disease syndromes in livestock animals and poultry. In this study, healthy as well as suspected sheep and goat populations from different regions of Kashmir valley were screened for the presence of *C. perfringens* toxinotypes. Our findings revealed that 110 (72.36%) of 152 sheep and 15 (60%) of 25 goats were positive for *C. perfringens* based on isolation and PCR amplification of 16S rRNA gene. The *C. perfringens* was isolated from both healthy and suspected enterotoxaemia cases. In accordance with our study, a lower occurrence of 24.13% of *C. perfringens* in sheep of Morocco [18] while as a higher prevalence of 100% of *C. perfringens* in sheep of Italy [19] has been recorded. Similarly, the prevalence of 59.62% and 75% of *C. perfringens* in sheep was reported in Andhra Pradesh, India [20]. The prevalence of 96.92% of *C. perfringens* in sheep and goats in Switzerland has been reported [21].

In this study, *C. perfringens* toxinotype D was more prevalent among lambs (56.16%) and kids (46.16%) than adult sheep (3.84%), respectively, while none of the *C. perfringens* isolates from adult goats belonged to toxinotype D. Our findings are in agreement with a research done in fattening lambs in the United Kingdom where enterotoxemia has been reported to be more prevalent in lambs aged between 3 and 8 weeks [22]. The authors attributed it to the heavy feeding and milking of lambs by ewes that are grazed on lush pastures. However, they also observed its higher prevalence in adult animals grazed on luxurious pastures. The spillover of the carbohydrate and protein-rich nutrients into the small intestine from the abomasum encourages rapid multiplication of organisms and production of ETX. The increased prevalence of *C. perfringens* Type D (21.65%) in lambs than healthy adult sheep (3.7%) has been reported in Andhra Pradesh, India [20].

The organized sheep rearing farms of the valley revealed the higher occurrence of toxinotype D (46.06%) in comparison to the unorganized sector (9.52%). This discrepancy could be attributed to lesser availability of the samples from lambs in comparison to the adults from the unorganized sources during the study period.

In this study, out of 110 sheep, 67 (60.90%) were found positive only for *cpa* toxin gene, thus belonged to toxinotype A, while the remaining 43 (39.09%) carried both *cpa* and *etx* toxin genes thus belonged to toxinotype D. None of these isolates possessed *cpb* or *cpi* toxin gene, indicating the absence of *C. perfringens* toxinotype B, C, or E. Similarly, in case of goats, 8 (53.33%) out of 15 isolates were detected as toxinotype A and the remaining 7 (46.66%) were toxinotype D. However, none of these isolates revealed the presence of *cpb* or *cpi* toxin gene, indicating the absence of *C. perfringens* toxinotype B, C, or E. These findings are in agreement with the observations reported from Italy where 84% of *C. perfringens* isolated from the lambs and kids in Italy is toxinotype A and the remaining 16% as toxinotype D, and none belonged to type B, C, or E [19]. In India, 69.29% prevalence of enterotoxemia from suspected sheep flocks and 39.71% from healthy sheep flocks has been reported [20]. Genotyping of the isolates from healthy flocks indicated the presence of toxinotype A and D to be 92.59% and 7.40%, respectively, and *C. perfringens* toxinotype A (67.01%), toxinotype D (21.65%) as well as type C (11.34%) from suspected flocks. Although toxinotype C has not been reported from sheep or goats in the present study, the presence of toxinotype B, C, or E cannot be ruled out owing to the fact the study being preliminary and based comparatively on small sample size.

In addition to four major toxin genes, two minor toxin genes *cpb2* gene and *cpe* gene were detected in 19 isolates of toxinotype A and D. In sheep, all *C. perfringens* toxinotype A isolates revealed the presence of species-specific *cpa* gene but none of the isolates harbored *cpb2* or *cpe*-gene. However, the prevalence of 42.8% of *cpb*2 gene among *C. perfringens* toxintypes A isolates in sheep in Iran has been reported by Jabbari *et al*. [23]. In contrast to this, *C. perfringens* toxinotype A isolates from goats carried *cpb*2-gene (12.50%) and *cpe*-gene (12.50%). The presence of *cpb2* gene in 4/21 (19%) of toxinotype A isolates from lambs and kids has been observed in Italy [19]. The *cpb2* gene and *cpe* gene can be carried by all *C. perfringens* toxinotypes [24].

In sheep, within *C. perfringens* toxinotype D isolates, 27.90% possessed *cpb2*-gene and 6.97% harbored *cpe*-gene in addition to the *cpa* and *etx*-genes. While out of seven *C. perfringens* toxinotype D isolates from goats 14.28% contained both *cpb2*-gene and *cpe*-genes. *Clostridium perfringens* enterotoxin and beta-2 toxin have been widely reported as potential contributors to *C. perfringens* related enteric diseases. The occurrence of *cpb2* gene in one out of two toxinotype D isolates in lambs and kids has been reported in Italy [19]. Similarly, in Iranian sheep, 69.25% of *C. perfringens* toxinotype D isolates possessed c*pb*2 gene [23]. While investigating *C. perfringens* toxinotype D in sheep and goats in Germany, it was observed that out of nine isolates from sheep five carried *cpe* gene while one out of three isolates from goats showed the presence of *cpe* gene [25].

## Conclusion

*C. perfringens* is highly prevalent among lambs and kids in Kashmir valley. The toxinotype A was more prevalent than that of toxinotype D in sheep and goats. Absence of toxinotypes B, C, and E in the present study does not indicate the absence of these toxinotypes in the sheep and goat population as the number of samples was less. The *C. perfringens* toxinotype D is more prevalent in organized than unorganized sector and mostly affects lambs and kids. The adult sheep and goats carry *C. perfringens* but cause the disease occasionally. The present work documents the prevalence, isolation and characterization of *C. perfringens* toxinotype D in sheep and goats of Kashmir valley. The present work also makes the local strains available for vaccine formulation to effectively control the menace in the state.

## Authors’ Contributions

SN carried out the study. SAW planned, designed and supervised the experiment, RP and SAA provided technical support, TAD, ZAD helped in collection of samples and ZAK, SH and PAD supported in drafting the manuscript. The authors are thankful to the Sheep Husbandry Department, Jammu and Kashmir, India, for providing positive control. All authors read and approved the manuscript.
